# Natural Killer Cells and Type 1 Innate Lymphoid Cells in Hepatocellular Carcinoma: Current Knowledge and Future Perspectives

**DOI:** 10.3390/ijms22169044

**Published:** 2021-08-22

**Authors:** Nicolas Jacquelot, Cyril Seillet, Fernando Souza-Fonseca-Guimaraes, Adrian G. Sacher, Gabrielle T. Belz, Pamela S. Ohashi

**Affiliations:** 1 Princess Margaret Cancer Centre, University Health Network, Toronto, ON M5G 2C1, Canada; adrian.sacher@uhn.ca (A.G.S.); pam.ohashi@uhnresearch.ca (P.S.O.); 2 Walter and Eliza Hall Institute of Medical Research, Parkville, Melbourne, VIC 3052, Australia; seillet@wehi.edu.au (C.S.); g.belz@uq.edu.au (G.T.B.); 3 Department of Medical Biology, University of Melbourne, Parkville, Melbourne, VIC 3010, Australia; 4 Diamantina Institute, University of Queensland, 37 Kent Street, Woolloongabba, Brisbane, QLD 4102, Australia; f.guimaraes@uq.ed.au; 5 Department of Immunology, University of Toronto, Toronto, ON M5S 1A8, Canada

**Keywords:** natural killer cells, innate lymphoid cells, ILC1, liver, hepatocellular carcinoma, cancer, immunotherapies

## Abstract

Natural killer (NK) cells and type 1 innate lymphoid cells (ILC1) are specific innate lymphoid cell subsets that are key for the detection and elimination of pathogens and cancer cells. In liver, while they share a number of characteristics, they differ in many features. These include their developmental pathways, tissue distribution, phenotype and functions. NK cells and ILC1 contribute to organ homeostasis through the production of key cytokines and chemokines and the elimination of potential harmful bacteria and viruses. In addition, they are equipped with a wide range of receptors, allowing them to detect “stressed cells’ such as cancer cells. Our understanding of the role of innate lymphoid cells in hepatocellular carcinoma (HCC) is growing owing to the development of mouse models, the progress in immunotherapeutic treatment and the recent use of scRNA sequencing analyses. In this review, we summarize the current understanding of NK cells and ILC1 in hepatocellular carcinoma and discuss future strategies to take advantage of these innate immune cells in anti-tumor immunity. Immunotherapies hold great promise in HCC, and a better understanding of the role and function of NK cells and ILC1 in liver cancer could pave the way for new NK cell and/or ILC1-targeted treatment.

## 1. Hepatocellular Carcinoma—Epidemiology, Pathophysiology, Risk Factors and Treatments

Hepatocellular carcinoma (HCC) is the most common form of primary liver cancer and accounts for ~90% of all cases [1]. Its incidence continues to rise globally [2]. Chronic viral hepatitis, alcoholic liver disease and non-alcoholic steatohepatitis are the main conditions that drive the development of HCC [1,3], which represents the fourth leading cause of cancer-related deaths worldwide [1,3]. HCC formation is a progressive and multi-step process requiring the combination of tumor-intrinsic and extrinsic factors to drive tumor formation [3,4]. These factors include dysregulation of the local microenvironment and chronic inflammation associated with altered genetic events (mutations, copy number variations and deregulating DNA methylome) that, together, promote tumorigenesis [3,4,5,6]. Family history of HCC is associated with increased risk of developing liver cancer, and males exhibit a higher prevalence than females in disease development [2]. Indeed, testosterone was shown to promote hepatocyte cell cycles while estradiol negatively regulates cell proliferation [7]. Exome sequencing of 243 liver tumors has revealed mutational signatures associated with pathogenic cofactors such as tobacco and alcohol consumption [5]. More than 25% of these tumors present genetic alterations that are drug-targetable using FDA-approved compounds, but this potential has yet to be translated into clinical practice [3,5,8]. More recently, a nationwide study performed on more than 50,000 patients has uncovered that low-dose intake of aspirin was significantly associated with a reduced risk of hepatocellular carcinoma and liver-related mortality compared with no aspirin, without increasing the risk of gastrointestinal bleeding [9]. Together, these advances in the comprehension of the pathophysiology of this disease will be likely translated in the coming years into reduced incidence and better clinical management of patients who suffer from chronic liver diseases and/or hepatocellular carcinoma.

Treatment options for this disease have increased considerably over the past decade [8]. Hepatic surgical resection and liver transplantation remains the standard of care treatment to cure HCC patients. With the increased precision of imaging systems, image-guided locoregional therapies are employed to treat early- and intermediate-stage HCC. Radiofrequency is the mainstay for local tumor ablation in non-surgical early-stage HCC, and transarterial chemoembolization is the preferred treatment for intermediate-stage HCC [10]. In addition, several systemic therapies have been approved over the past few years for the management of unresectable HCC, dramatically changing the therapeutic landscape for patients with unresectable advanced disease. Three categories of drugs, alone or in combination, are currently used to treat unresectable HCC: (i) immune checkpoint blockers (ICB), (ii) tyrosine kinase inhibitors (TKI) and (iii) monoclonal antibodies. Recently, the combination of atezolizumab (anti-programmed death-ligand 1 (PD-L1) antibody) with bevacizumab (anti-vascular endothelial growth factor (VEGF) antibody) has demonstrated increased progression-free and overall survival of HCC patients compared with the TKI sorafenib alone [11]. This has led to the emergence of atezolizumab–bevacizumab as the standard initial therapy in advanced HCC. However, sorafenib [12] and lenvatinib [13] remain standard-of care treatments in the second-line setting, while regorafenib [14], cabozantinib [15] and ramucirumab [16] may be employed upon subsequent progression. Programmed death-1 (PD-1) inhibitors (e.g., nivolumab, pembrolizumab) and a combination of PD-1 with cytotoxic T lymphocyte-associated antigen-4 ( CTLA-4) inhibitors (nivolumab-ipilimumab) have also demonstrated activity in advanced HCC. Despite these significant advances, the clinical benefit remains modest and many patients relapse within 6 to 8 months after therapy initiation [11]. Additional therapies involving the combination of multiple ICB together or ICB with TKI are currently being tested in clinical trials [17,18]. The results of these clinical trials will probably further augment the oncological armamentarium for HCC.

Unresectable HCC remains an area of unmet medical need, with limited treatment options after progression on atezolizumab–bevacizumab. Thus, alternative approaches must be identified to provide new therapeutic options for HCC patients. Given the promising results revealed by the use of ICB in clinical trials [11,19], the tumor microenvironment, in particular the immune system, appears to be an attractive target. Both innate and adaptive immunity are necessary to control cancer development and progression and impact treatment responses [20,21], and HCC is no exception [22,23,24,25,26,27,28,29,30,31,32]. Here, we review recent advances in our understanding of the role and function played by NK cells and ILC1 in HCC. We describe NK cells and ILC1 ontogeny and phenotype as well as the differences that exist between human and mouse ILCs that may pose issues while studying their function using pre-clinical HCC models. Furthermore, we report their described effector functions and prognostic values in HCC and how their key features could be harnessed to develop potential new therapies.

## 2. Innate Lymphoid Cells—Hepatic NK Cells and ILC1

Innate lymphoid cells are evolutionary conserved innate immune cells that play essential homeostatic function in tissues [33,34]. This family is composed of five subsets, namely NK cells, ILC1, ILC2, ILC3 and lymphoid-tissue inducer cells (LTi) [35]. ILCs are the innate counterpart of the adaptive lymphocytes and provide a first line of defense to protect epithelial barriers [36,37]. The absence of antigen-specific receptors allows ILCs to respond quickly to any immunological threat, including virus and bacterial infections, as well as neoplastic transformation [38]. In contrast, these innate cells are also easily prone to dysregulation and over-activation, which can act to sustain inflammation-driven autoimmune disorders [39]. ILCs are equipped with a wide range of receptors and ligands at their surface, allowing them to engage with other cell types in their microenvironment [40,41,42]. In addition to cell–cell interactions, ILCs communicate with other cells through the production of a large array of cytokines and chemokines [35] which, collectively, identify these innate cell subsets as key orchestrators of homeostatic immunity and inflammatory responses.

Discovered more than 45 years ago, conventional NK (cNK) cells were first described as an effector lymphoid population with the capacity to lyse tumor cells [43,44,45,46,47]. These circulating innate cells patrol throughout the body to detect and eliminate infected or transformed cells. They represent 5–15% of blood lymphocytes in humans and 2–5% in mice. They are characterized by the expression of the natural cytotoxicity triggering receptor NKp46 (or Ncr1) in mice and humans, or CD56 in human, as well as by the expression of additional activating and inhibitory receptors, which further regulate their activity and function [48,49]. Peripheral immature and mature cNK cells can be subdivided based on CD11b and CD27 expression in mice [50,51,52], or CD56 and CD16 in humans [48,53,54]. All cNK cells express the transcription factors Eomesodermin (Eomes) and T-box Transcription Factor 21 (T-bet, encoded by *Tbx21*), which drive the production of key cytokines and cytotoxic molecules. These include granzyme, perforin and interferon (IFN)-γ [49]. As early as 2005, before the appreciation of the diversity within type 1 ILCs, Takada et al. [55] described, for the first time, a population of liver resident NK cells (ILC1) expressing NK1.1, NKp46 and TNF-related apoptosis-inducing ligand (TRAIL) but not CD49b (DX5). These cells are present in the liver of fetal and neonatal mice and persist in adults, and their presence partly relies on IFN-γ expression [55]. Over the following years, additional markers distinguishing ILC1 from hepatic cNK cells were reported (Table 1). cNK cells express DX5 and Eomes, while ILC1s express CD49a, TRAIL, CD200R, C-X-C Motif Chemokine Receptor (CXCR) 6 and CD127 [56] (Table 1). In parallel, investigations in human samples have revealed the presence of liver ILC1s, which are identified as CD45^+^lineage^−^CD127^+^CD16^−^ NK group 2 member A (NKG2A)^−^NKp44^−^CD117^−^ [57]. Similar to mouse hepatic ILC1, human ILC1 express T-bet but lack Eomes expression [57]. However, the CD56 status of ILC1 was not reported. Within the CD56^+^CD3^−^ population, typically defining cNK cells, an equal proportion of CD56^bright^ and CD56^dim^ are found in the liver, mirroring proportions found in lymph nodes (75% and 25%, respectively) and contrasting with the proportions found in blood (5–10 and 90–95%, respectively) [58,59]. The CD56^bright^ subset express features of tissue-residency such as CXCR6 and CD69 and thereby resembling mouse ILC1. This long-lived subset also expresses high levels of Eomes but low levels of T-bet [60,61,62,63,64,65]. Given these specific properties, these cells are considered to be liver-resident (lr) NK cells distinct from cNK cells, which circulate throughout the body. In addition, within this CD56^bright^ lrNK cell subset, a small population express CD49a and T-bet, but lack Eomes expression [66], reminiscent of mouse hepatic ILC1. Yeang et al. [67] reported a CD56^bright^CD49e^−^ lrNK cell subset that expresses CXCR6 and Eomes, but lacks T-bet [67], a phenotype further confirmed using high-dimensional flow-cytometry analyses [68]. Thus, multiple tissue-resident NK cells and ILC1 subsets may exist in human liver that contrast with the two well-delineated populations identified in mice.

Over the past four years, a number of single cell RNA sequencing studies have profiled the mouse and human liver, in health and disease, shedding light on the potential interactions that might occur between immune and parenchymal cell subsets at the organ level [74,75,76,77,78,79,80,81,82,83]. While this approach holds great promise, the identification of the different hepatic NK cell subsets and ILC1 remains limited [84]. This is likely due to the relatively low number of cells that have been profiled. Indeed, the analysis of up to several million immune cells by flow-cytometry are needed to identify rare immune cell populations such as ILC1 in human liver, where, in single cell RNA sequencing analyses, the number of cells profiled rarely goes over 100,000. As a consequence, most of the studies detected only one NK cell cluster. Only two studies, led by Ramachandran et al. [76] and Zhao et al. [78], have identified several NK cell subsets. These include the liver resident and conventional circulating NK cells. They observed that lrNK cells have increased expression of *CD160*, *T cell immunoglobulin and ITIM domain (TIGIT)*, *Killer Cell Lectin Like Receptor C1* (*KLRC1)*, *Granzyme K* (*GZMK)*, *X-C Motif Chemokine Ligand 1* (*XCL1)* and *XCL2* compared to cNK cells. Conversely, cNK cells displayed increased cytotoxic functions through augmented expression of *GZMB*, *GZMH*, *Granulysin* (*GNLY*) and enhanced *FCGR3A* (coding for CD16), *NCR3*, *CD226*, and *KLRG1* expression compared to lrNK cells [76,78,84]. Collectively, there is a need for the aggregation and integration of multiple datasets in which the authors have identified NK cell clusters to increase the number of profiled immune cells and refine analyses aiming to identify additional NK cell subsets and their specific transcriptional profiles. Alternatively, the profiling of the liver CD3^−^CD56^+^ subsets, as previously performed for the blood and spleen [54], using single cell RNA sequencing would definitively provide new insights into the complexity and diversity within the hepatic NK cells. Ultimately, identified markers at the transcriptional level that delineate hepatic NK cell and ILC1 subsets and potentially lead to the description of new subpopulations must be validated at the protein level. A better understanding of NK cells and ILC1 subsets in liver would open up new possibilities to target these immune cell populations in HCC.

## 3. Diversity within Hepatic NK Cell and ILC1 Subsets

**Figure 1:**
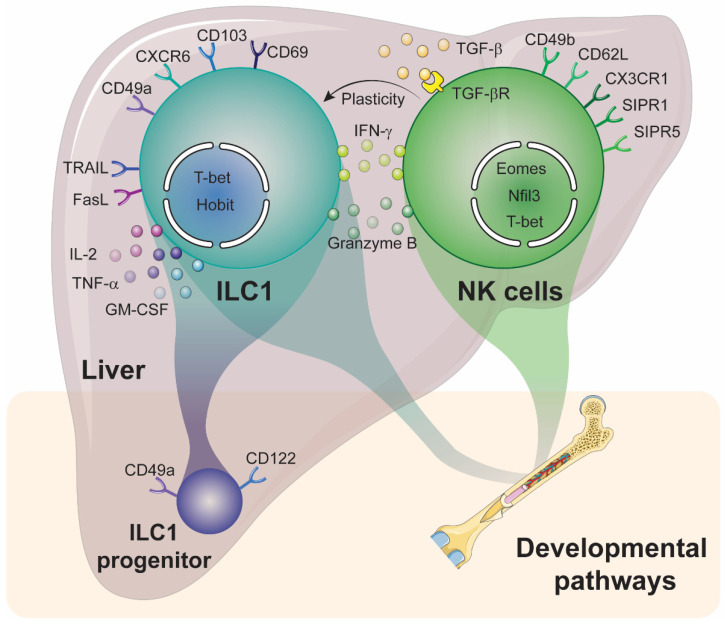
Specificities of hepatic NK cells and ILC1. Liver ILC1s can be generated from a local or from bone marrow-derived progenitors. The different surface markers expressed by liver ILC1 and NK cells at steady-state are shown, together with the transcription factors that regulate their respective development. Under transforming growth factor-β (TGF-β) signaling, NK cells can upregulate ILC1 markers such as TRAIL or CD49a.

Despite obvious similarities, numerous preclinical studies have shown that liver NK cells and ILC1 differ in many aspects [85,86]. These include their developmental pathways, transcriptional programs, function and their plasticity (Figure 1). Indeed, their ability to transdifferentiate into one another further increases the complexity within this group 1 ILC.

**Developmental pathways.** The transfer of bone marrow into lethally irradiated recipient mice allows the reconstitution of hepatic NK cells but very few liver ILC1 [56]. However, fetal liver transfer gives rise to both liver NK cells and ILC1 [56], indicating that liver ILC1, contrary to NK cells, mainly develop from local hematopoietic progenitors, highlighting an early divergence in their ontogeny. Until recently, this progenitor has not been known. Recent investigations made by Bai et al. [87] have revealed that fetal liver hematopoietic stem cells (HSCs) were enriched in a lineage negative population that express CD122 and CD49a and differentiate into ILC1 but not cNK cells, confirming the observations first made by Takeda et al. [55], who found that IFN-γ-producing ILC1 sustains the proliferation of the progenitors and their differentiation into mature hepatic ILC1 [87]. The disruption of this IFN-γ signaling pathway selectively reduced the frequency of hepatic ILC1 but not cNK cells. Conversely, other groups have observed that the adoptive transfer of bone marrow ILC progenitors into sub-lethally irradiated recipient *Rag2^−/−^Il2rg^−/−^* mice gives rise to liver ILC1 but not cNK cells [88,89]. These discrepancies suggest that, under pro-inflammatory conditions such as sublethal irradiation, liver ILC1 can also differentiate from bone marrow progenitors to quickly expand the pool of tissue-resident ILC1 needed to fight potential infection.

**Transcriptional programs.** The development of hepatic ILC1 and cNK cells relies on distinct transcriptional programs that involve four key transcription factors. These are Eomes, T-bet, zinc finger protein 683 (ZNF683 or Hobit) and nuclear factor, interleukin 3 regulated (Nfil3) (Figure 1). cNK cells necessitate *Eomes* expression for their development [90], while ILC1 progenitors require *Tbx21* (encoding for T-bet) expression for their differentiation into mature hepatic ILC1 [87], further confirming previous observations [69,70] (Table 2). It is also important to note that enforced expression of Eomes induces NK cell features amongst ILC1 [71,91] and, reciprocally, T-bet overexpression promotes ILC1 development over cNK cells [70]. The expression of the transcription factor Hobit, which instructs a tissue residential program in lymphoid cells, is necessary for liver ILC1 development [73]. In *Hobit* deficient mice, hepatic ILC1 are absent, whereas the frequency of liver cNK cells are not impacted. This is due to the absence of Hobit expression in this cell type [73]. Finally, a fourth transcription factor called *Nfil3* is differentially required for liver ILC1 and cNK cell development. While *Nfil3* deficiency in early progenitors strongly impacts the development of all ILC subsets, including cNK cells [92,93,94,95], it does not impact or only slightly reduced the frequency of ILC1 in the liver [69,71] or in the salivary glands [96]. In contrast, loss of *Nfil3* strongly impaired the development of mucosal intraepithelial ILC1 [97], highlighting the heterogeneity within this group 1 ILCs, in mice [85].

**Tissue residency.** NK cells circulate throughout the body, whereas liver ILC1 are tissue resident cells, as demonstrated using parabiotic mice, that establish blood chimerism through joint circulation [56,98]. This migration and tissue retention is mediated by the expression of chemokine receptors, integrins and selectins. Transcriptional analyses of liver ILC1 and cNK cells have revealed distinct patterns of expression for these molecules. ILC1 express CXCR6, CXCR3, CD103, CD49a and CD69, whereas cNK cells rely on the expression of CD62L, C-X3-C Motif Chemokine Receptor 1 (CX3CR1), C-C Motif Chemokine Receptor 9 (CCR9), Sphingosine-1-phosphate receptor (SIPR) 1 and SIPR5 [56,70,71] (Figure 1). In humans, lrNK cells express CXCR6, CD69 and CCR5, while cNK cells rely on the expression of SIPR1, CCR7, L-selectin and CX3CR1 for their migration or tissue retention [60,61]. Thus, the differential expression of chemotactic receptors likely contributes to their specificity towards tissue distribution and residency.

**Function.** Since their discovery, cNK cells are considered as cytotoxic innate immune cells, whilst ILC1 are deemed helper innate cells due to their capacity to secrete multiple cellular mediators and pro-inflammatory cytokines. However, given the heterogeneity amongst ILC1s, differences in ILC1 function undoubtedly exist. Indeed, contrary to intestinal ILC1s, liver ILC1s express the cytotoxic molecules granzymes and perforin, akin to cNK cells [69]. Despite a significant reduction in IFN-γ production and degranulation, liver ILC1s can effectively kill target cells in vitro. However, they have specific properties, very distinct from cNK cell features. Liver ILC1 produce high amounts of TNF-α, IL-2 and GM-CSF and express the ligands for Fas and TRAIL receptors [69,70] (Figure 1). In humans, tissue-resident lrNK cells (CD56^bright^) have increased expression of activating receptors NKp44, NKp46 and NKG2D and enhanced expression of TRAIL, but reduced capacity to produce IFN-γ, granzyme B and perforin compared to CD56^dim^ or CXCR6^−^ NK cells [62,63]. However, when exposed to target cells, they have enhanced degranulation capacities compared to the CD56^dim^ subset [63,64]. Collectively, NK cell and ILC1 subsets in the liver have distinct functions that would impact their ability to mount an immune response against HCC, likely influencing disease outcomes.

**Plasticity.** Mature ILCs originate from distinct developmental pathways, although they can adopt a different phenotype resembling another ILC population when stimulated with the appropriate cocktails of cytokines and other molecules [99,100]. In the presence of TGF-β, particularly abundant in the tumor microenvironment, NK cells (CD49a^−^CD49b^+^) can transdifferentiate into intermediate ILC1 (CD49a^+^CD49b^+^), sharing features from both ILC1 and NK cells, and into ILC1 (CD49a^+^CD49b^−^) [101,102] (Figure 1). On tumor progression, NK cells progressively upregulate CD49a and TRAIL and downregulate Eomes expression. Simultaneously, they lose their capacity to produce IFN-γ and lyse tumor cells and fail to limit tumor growth and metastases. Together, these studies have revealed the critical role of TGF-β signaling on NK cell transdifferentiation into ILC1-like cells in the tumor microenvironment, negatively impacting tumor prognosis [101,102]. In the liver, TGF-β is abundantly produced by both immune and non-immune cells, promotes tissue fibrosis, and is particularly abundant in tumors [65,103,104,105,106]. In HCC, high levels of TGF-β are found in the plasma of patients, which is associated with a decrease of NK cell function and reduced survival compared to patients with lower levels of plasmatic TGF-β [107]. Harmon et al. [65] found that the culture of lrNK cells in vitro induced the upregulation of T-bet expression, while levels of CXCR6 progressively decreased [65]. When cultured in liver-conditioned media, T-bet upregulation is prevented [65]. Similar observations were made when peripheral blood NK cells were cultured in liver-conditioned media [65], suggesting that a soluble mediator present in the liver inhibits T-bet expression in lrNK cells. They identified TGF-β as being responsible for the downregulation of T-bet, thus participating in the maintenance of tissue residency of these lrNK cells. Together, observations made by Harmon et al. [65] are complementary to previous pre-clinical work concerning the role of TGF-β in maintaining a tissue residency program in lymphocytes. It remains to be seen whether a similar mechanism instructs tissue residency in hepatic ILC1. In mice, TGF-βR2 deletion in NKp46 expressing cells abrogates the ILC1 phenotype associated with the upregulation of TRAIL/CD49a in the salivary glands [108]. However, the TGF-βR2 deficiency had a minimal impact on liver ILC1 [108], suggesting that other pathways, such as activin-A (another TGF-β superfamily member) [109], may also guide their differentiation. The transdifferentiation of NK cells into ILC1 would likely occur in HCC, but, to date, this remains to be fully determined. Such findings would certainly impact NK cell- or ILC1-directed immunotherapy development and efficacies [110].

## 4. Liver Immunity—An Immunotolerant Organ

The immune system is key to fighting against tumor cells and preventing cancer development and progression but, paradoxically, also contributes to HCC promotion as a key extrinsic factor of inflammation-driven carcinogenesis. Innate immunity is the first line of defense against viral infections and tumor development [111]. While NK cells are mainly associated with anti-tumor responses, the role and functions of ILC1 are more elusive [38,112]. In the liver, innate lymphocytes, particularly NK cells and ILC1, account for a large proportion of lymphocytes [56,71,111] and participate in the maintenance of organ homeostasis. In physiological conditions, the liver has to maintain an immunosuppressive environment to avoid excessive inflammation and damage as a result of the high bacterial load and food-derived antigens coming from the intestine [113]. Immune and non-immune cell types in the liver participate in the maintenance of a tolerogenic environment using distinct and complementary mechanisms [113,114,115,116,117,118,119]. These notably include the expression of PD-L1 and the release of immunosuppressive mediators such as IL-10, TGF-β, nitric oxide or prostaglandins, which, collectively, suppress the immune response locally. Thus, despite the specific properties of group 1 ILCs and their ability to detect and eliminate virus-infected and tumor cells [49,120], this unique immunological microenvironment in which they evolve [84,111,113,114] likely impacts the function of NK cells and ILC1 and their role in influencing immune responses, disease prognosis and treatment efficacies.

## 5. NK Cells in HCC

Since the 1980s, seminal studies have demonstrated the critical role of NK cells in tumor immune surveillance. In mice, the absence of NK cells by using depleting antibodies or engineered mouse models deficient in NK cells or NK cell cytotoxicity resulted in increased tumor development and metastases [121,122,123,124]. Similarly in humans, healthy individuals with peripheral mononuclear cells harboring low cytotoxic function have increased incidence rates of developing cancers [125]. In addition, low NK cell activity was found in cancer patients and their relatives associated with a high familial incidence of cancer [126,127], indicating that NK cells and their cytotoxic function may play a role as early as in the initial steps of tumor initiation as well as in the control of tumor development and metastases.

**Decreased NK cell frequency and numbers in HCC patients.** Lower NK cell numbers, reduced NK cell activity or the absence of an NK cell signature in HCC are all associated with poor patient outcomes [128,129,130,131,132,133,134]. Several studies have revealed reduced circulating NK cells harboring impaired cytotoxic functions in HCC patients [135,136,137]. Patients suffering from non-alcoholic liver cirrhosis have increased HCC incidence rates when NK cell activity is reduced [135], suggesting that NK cell function is critical during the initial stages of cancer development. Furthermore, Cai et al. [136] found a decrease of the proportion of total NK cells in the blood of HCC patients, irrespective of disease stage, which was associated with an enrichment of the CD56^bright^ subset compared with healthy donors. The expression of granzyme, perforin and IFN-γ were all reduced, resulting in a reduced ability to lyse target cells in vitro [136]. The analyses of normal and tumor hepatic tissues have revealed a strong decrease of intratumoral NK cells in tumors compared to non-tumoral tissues [133,134,138]. Regarding the distribution of the different NK cell subsets within tumors, Zecca et al. [138] have reported an enrichment in CD56^bright^ NK cells, associated with decreased expression of CXCR6 and Eomes and an increased frequency of T-bet^+^ and CD49a^+^ CD56^bright^ NK cells. In contrast, Sun et al. [139] observed a decreased frequency of CD56^bright^ cells with a corresponding increase of CD56^dim^ NK cells compared to peri-tumoral tissues. Similar to blood NK cells, the function of intratumoral NK cells is impaired due to the reduced expression of cytotoxic molecules and pro-inflammatory cytokines [134,136,138], indicating that several immunosuppressive mechanisms exerted by the tumor microenvironment are in place to prevent NK cell anti-tumor function. Treatment with recombinant IL-2 or IFN-γ increased NK cell function and activity; however, the cytotoxic activity of NK cells isolated from HCC patients against target cells remains lower compared to patients suffering from other liver-related diseases [132]. Together, this confirms that NK cells are functionally dysregulated in HCC [140].

More recently, Sun et al. [24] have observed that the accumulation of intratumoral CD49a^+^ lrNK cells in human HCC correlated with poor prognosis. These cells express higher levels of immune checkpoint molecules compared to CD49a^−^ NK cells and include TIGIT, CD96 and PD-1 [24]. In addition, the CD49a^+^ subset expresses lower levels of NCR NKp30 and NKp46 as well as other activating receptors compared to CD49a^−^ NK cells. The accumulation of lrNK cells was associated with increased tumor diameter, reduced disease free and overall survival of HCC patients [24], potentially indicating a tumor-promoting function of these innate cells. However, we do not know whether the accumulation of these cells is the cause or the consequence of the increased tumor burden, and this remains to be determined.

## 6. NK Cell Biology

NK cells are one of the main actors that efficiently detect and kill tumor cells. Once activated, owing to the expression of activating receptors, NK cells kill target cells via the release of effector molecules forming pores. In contrast, to protect healthy cells from our body, NK cells are also equipped with multiple inhibitory receptors to avoid non-specific targeting and auto-immune disorders.

**Figure 2:**
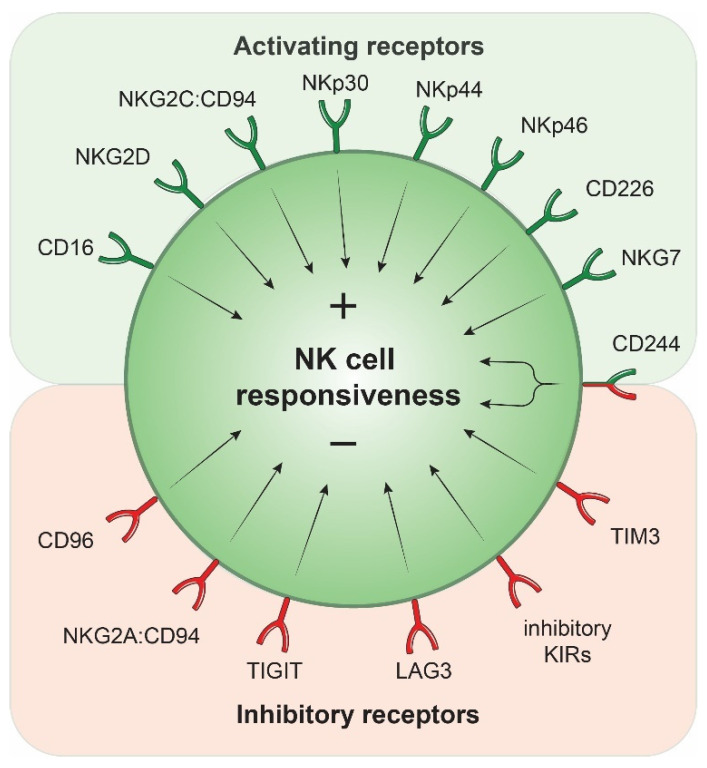
NK cell activating and inhibitory receptors. NK cell responsiveness is modulated by the engagement of activating or inhibitory receptors at the cell surface. TIM3, T cell immunoglobulin and mucin domain-containing protein 3.

**NK cell activating receptors.** NK cell activity is determined by a fine balance between activating and inhibitory receptors, recognizing ligands expressed by immune and non-immune cells, which, together, dictate NK cell responsiveness [49,141,142] (Figure 2). Since the 1980s, many activating and inhibitory receptors have been described. Some activating receptors include CD16 [143], NKG2C:CD94 [144,145], NKG2D [146,147], NCR1 (NKp46, CD335) [148,149], NCR2 (NKp44, CD336) [150], NCR3 (NKp30, CD337) [151], CD226 (DNAX Accessory Molecule-1 or DNAM-1) [152], CD244 (2B4) [153,154] or natural killer cell granule protein 7 (NKG7) [155]. In most cases, the binding of these receptors to their ligands stimulates NK cell function. CD16 expression allows NK cells to bind IgG antibodies, eliciting the recognition and killing of antibody-coated target cells [143,156,157]. The heterodimer NKG2C:CD94 binds to the non-classical major histocompatibility complex (MHC) class I molecule HLA-E in humans or Qa-1 in mice [158,159] and is highly expressed on a subset of NK cells found upon infection with the human cytomegalovirus [160]. The activating receptor NKG2D associates with the adaptors DNAX-activation protein (DAP)10 or DAP12 to transmit signals upon binding to its ligands, which include the MHC class I chain–related protein A (MICA), MICB, or UL16 binding protein (ULBP) proteins in humans and H60, RAE and mouse UL16-binding protein-like transcript 1 (MULT1) families in mice [161,162,163]. While the expression of these proteins at the surface of normal cells is limited, these ligands are widely expressed at the membrane of stressed cells such as tumor cells [146,147,161], allowing recognition and tumor cell lysis by NK cells. Regarding the NCR family, many ligands have also been described [164]. Some examples include the complement factor P [165], platelet-derived growth factor-D (PDGF-DD) [166] or B7-H6 [167]. The activating receptor CD226 triggers NK cell activation upon binding to its ligand CD155 or CD112 [168,169,170]. One of the most studied receptors is probably CD244. It is a member of the SLAM family, a group of immunoregulatory receptors expressed by multiple immune cell types, including NK cells. CD244 binds to CD48, a molecule expressed constitutively at the surface of many immune and non-immune cell subsets [171], including intratumoral hepatic monocytes [134]. Finally, NKG7 is an integral membrane protein that favors NK cell exocytosis through the promotion of CD107a translocation to the cell membrane and the killing of target cells [155,172,173]. Collectively, the engagement of these activating receptors by their ligands promotes NK cell activity and lysis of the target cells.

**NK cell inhibitory receptors.** To counterbalance the function of these activating receptors, NK cells also express a myriad of inhibitory receptors. These notably include CD96 [169], TIGIT [169], NKG2A:CD94 [145,174,175,176], Lymphocyte Activating 3 (LAG3) [177,178] or the inhibitory killer cell immunoglobulin-like receptors (KIRs) (inhibitory Ly49 receptors in mice) [141,179] (Figure 2). The heterodimer NKG2A:CD94, KIRs and LAG3 recognize MHC-Class I/II molecules expressed abundantly on normal cells, which inhibits NK-cell function [141,158,175,176,180]. Tumor cells, to avoid immune recognition by adaptive immune cells, often downregulate MHC Class I expression, which should then bias signals towards NK cell activation and tumor cell killing. In contrast to CD226, the inhibitory receptors CD94 and TIGIT bind to CD155 and CD112, respectively, and impair NK cell activity [169]. Some receptors can be either activating or inhibitory, further increasing the complexity of the NK cell biology. This is the case for CD244, where the dichotomous function is dictated by the levels of expression of the intracellular adaptors that mediate signal transduction [134,153,154,181]. Collectively, this complex system, involving many receptor-ligand interactions, directly dictates the capacity of NK cells to mediate their function and represents a potential targeting approach to reverse NK cell dysfunction in cancer.

## 7. NK Cell Dysfunction in HCC

Tumor cells and other tumor-promoting cells have put in place strategies to disrupt these receptor-ligand interactions, aiming to escape NK cell-mediated killing [182,183]. These include (i) the downregulation/upregulation of activating/inhibitory receptors, (ii) downregulation/upregulation/shedding of the corresponding ligands that activate/inhibit NK cells and (iii) the accumulation of microenvironment-derived immunosuppressive factors that inhibits NK cell proliferation and function [182,183].

**NK cell intrinsic mechanisms.** Recent investigations have demonstrated that, in HCC, NK cells upregulated the inhibitory receptors NKG2A, TIM3 and CD96 and downregulated TIGIT, NKG2D, NKp30, NKp46, sialic acid binding Ig-like lectin 7 (Siglec-7) and CD160 expression, which was associated with the impairment of NK cell function and poor prognosis [106,138,139,184,185]. However, some discrepancies may exist between studies as Zecca et al. [138] reported increased expression of NKp30 on intratumoral NK cells compared with hepatic NK cells from non-tumoral tissue. An increase in CD96 expression on NK cells was inversely correlated with IFN-γ production. The transcriptional analysis of CD96^+^ NK cells has revealed an enrichment in the expression of the inhibitory receptors *PDCD1* and *LAG3* (coding for PD-1 and LAG3, respectively), as well as in the expression of the anti-inflammatory cytokine IL-10. This immunoregulatory phenotype was associated with a diminution of the expression of the cytotoxic molecules *PRF1* and *GZMB* and the activating receptors *NCR2*, *NCR3*, *CD226*, *CD244* and *NKG7*. This exhausted NK cell phenotype was associated with reduced disease-free and overall survival [139]. Similarly, increased NKG2A expression on intratumoral NK cells was associated with reduced disease-free and overall survival [184]. Pre-clinical work has shown that sustained NKG2A expression on liver NK cells and reduced functionality were partially dependent on IL-10 [186]. In fact, the blockade of the IL-10/IL-10R signaling using antibodies specifically reduced NKG2A expression on hepatic NK cells but not splenic NK cells [186], indicating the role played by this immunosuppressive cytokine in NK cell dysfunction. In contrast, intratumoral downregulation of CD160 expression, a receptor involved in the promotion of NK cell function, was associated with a poor clinical outcome in HCC [106]. Peripheral CD160^+^ NK cells displayed increased levels of expression of NCRs, NKG2D and CD244, as well as TIGIT and increased IFN-γ production [106]. Intratumoral NK cells have reduced CD160 expression and tumor-infiltrating CD160^+^ NK cells showed signs of exhaustion. They exhibited reduced IFN-γ production and increased inhibitory receptor expression compared with their CD160^+^ peri-tumoral counterpart. Despite this functional impairment, high infiltration of HCC tumors with CD160^+^ NK cell was associated with both increased disease-free and overall survivals [106]. Similar to CD160, Siglec-7 expression on NK cells was decreased. The Siglec-7^+^ NK cells have increased NKp30 and NKp46 expression associated with augmented TNF-α expression [185], suggesting that they may play a positive role in HCC prognosis. IL-1R8, a member of the IL-1R family, inhibits NK cell maturation and anti-tumor function [187]. Its genetic deletion reinstates NK cell-mediated anti-tumor immunity in liver cancer [187], providing a rational to target IL-1R8 in HCC. Mantovani et al. [188] have observed a decreased expression of NKp30 and NKG2D associated with increased membrane expression of TIM-3 on circulating NK cells isolated from HCC patients compared to healthy controls. Three major isoforms of NKp30 have been described, namely NKp30a, NKp30b and NKp30c [189]. While NKp30a and NKp30b isoforms are associated with pro-inflammatory features, including the production of IFN-γ and TNF-α, NK cells, which predominantly express NKp30c, secrete high levels of IL-10 and present an immunoregulatory phenotype associated with reduced patient survival and response to treatment in many tumor types and auto-immune diseases [189,190,191,192,193,194]. In HCC, NK cells have decreased expression of NKp30a and NKp30b variants resulting in a decreased NKp30ab/NKp30c ratio, shifting NK cell function towards an immunoregulatory phenotype [188]. Furthermore, high levels of B7-H6 expression and increased sB7-H6 are found in the tumors and plasma of HCC patients, respectively, positively correlating with disease stage and driving NKp30 expression downregulation on NK cells [188]. In addition to a modification of the expression of activating and inhibitory receptors, intratumoral NK cells also exhibit an altered secretome profile and metabolism [138,195]. Particularly, tumor-infiltrating NK cells expressed higher levels of VEGF, IL-8, matrix metallopeptidase 9 (MMP9), placental growth factor (PlGF) and angiogenin [138], which, collectively participate in tumor angiogenesis, tumor microenvironment remodeling and tumor growth. In addition, tumor-infiltrating NK cells have fragmented mitochondria through hypoxia-induced activation of mTOR-Drp1 signaling, impairing NK cell survival and worsening disease outcomes [195]. Importantly, the restoration of NK cell mitochondrial metabolism was associated with increased cytotoxic function. Furthermore, reduced tumor size was observed in mouse models of human HCC in which metabolically efficient NK cells were adoptively transferred [195]. Collectively, these findings indicate that tumor-infiltrating NK cells in HCC are broadly dysregulated, which impairs their ability to eliminate tumor cells.

**NK cell extrinsic mechanisms.** The tumor microenvironment modulates NK cell function. The expression of ligands for inhibitory receptors or the secretion of soluble mediators by immune and non-immune cells inhibit NK cell function. It is well demonstrated that cancer-associated fibroblasts (CAF), myeloid cells and regulatory T cells (Tregs), in most cases, promote tumor growth and metastasis through diverse mechanisms [196,197,198,199,200,201,202]. In HCC, CAFs inhibit NK cell function. The co-culture of NK cells and CAFs reduced NK cell expression of the activating receptors associated with decreased NK cell function [203]. These include the downregulation of NKG2D, CD226 and NKp30, as well as a decrease of Granzyme B, Perforin, TNF-α and IFN-γ. In this work, they identified IDO and prostaglandin (PG)E2 as responsible for NK cell dysfunction, and the use of specific inhibitors against these pathways restored NK cell phenotype and activity [203]. These results indicate that CAFs are able to inhibit NK cell function through the secretion of specific immunoregulatory molecules. Monocyte/macrophages also promote NK cell dysfunction in HCC, resulting in decreased NK cell proliferation and activity and reduced survival [128,134,204]. In vitro cultured NK cells with an agonistic antibody targeting CD244 impaired NK cell IFN-γ and TNF-α production [134], further highlighting the dual role played by this receptor. Furthermore, monocytes drive NK cell dysfunction through the abnormal engagement of NKp30, resulting in reduced IFN-γ production and defects in tumor cell lysis [128]. The adoptive transfer of cytokine-induced NK cells resulted in an increased myeloid-derived suppressor cell (MDSC) recruitment within tumors that impaired anti-tumor immunity through the expression of Arginase 1 and inducible nitric oxide synthase (iNOS), causing NK cell dysfunction [204]. The pharmaceutical targeting of MDSC end products iNOS or arginase 1, or systemic treatment with a phosphodiesterase V (PDE5) inhibitor, suppressed MDSC accumulation and function in HCC, reinstating an important role for NK cells in anti-tumor immunity [204]. IL-10 producing tumor-associated macrophages (TAM) also accumulate in HCC and negatively correlate with granzyme B^+^ NK cells [205], indicating a possible inhibition of intratumoral NK cell function by TAM. Furthermore, liver macrophages (Kupffer cells) also secrete IL-10 [206], which may further suppress NK cell function in the context of HCC. Finally, a growing body of work indicates that Tregs directly or indirectly inhibit NK cell anti-tumor function [207,208]. The secretion of IL-8, IL-10 and TGF-β acts on NK cells and tumor cells and induces MICA/B shedding, the downregulation of MHC-Class I and the impairment of NK cell activity [208]. Furthermore, Tregs can modulate NK cell activity in a cell–cell dependent manner, mediated by CTLA-4, further damaging NK cell function and activity in HCC [208].

The stress-inducible NKG2D ligands are expressed at the membrane of tumor cells across many cancer types [147] and stimulate anti-tumor immunity [162]. Tumor cells may release soluble forms of NKG2D ligands to escape NK cell-mediated recognition and killing. Membrane bound ligands are shed upon enzymatic cleavage by the members of the ADAM (a disintegrin and metalloprotease) protease family [209,210,211,212,213]. In HCC, high MICA expression in the tumor microenvironment was associated with increased disease-free and overall survivals [214,215], whereas high soluble MICA (sMICA) found in the serum of patients was associated with larger tumor size and reduced overall survival [216,217,218]. NKG2D expression on NK cells was inversely correlated with the serum concentration of sMICA [216], suggesting that the engagement of MICA induces NKG2D downregulation on NK cells, leading to NK cell dysfunction in patients with high levels of sMICA. These results are reminiscent of the findings made by Groh et al., who observed decreased NKG2D expression on CD8^+^ T cells infiltrating MIC^+^ tumors [219]. Similarly, NKG2D downregulation is observed on NK cells in mice overexpressing the NKG2D ligands Rae-1ε, increasing the susceptibility of mice to tumorigenesis [163]. Mechanistically, the binding of MIC triggers NKG2D endocytosis and degradation, negatively impacting T cell and NK cell responses [163,219]. In vitro cultures of NK cells with serum containing high levels of sMICA induced the downregulation of NKG2D expression associated with reduced IFN-γ secretion [220] and the impairment of NK cell cytotoxicity and killing capacities against MICA-expressing target cells [218]. Similarly, high concentration levels of soluble ULBP1 in the serum of HCC patients are associated with reduced overall survival [221]. These findings, collectively, indicate that NKG2D ligands represent critical therapeutic targets to reinstate NK cell anti-tumor function.

## 8. Therapeutic Intervention

**Figure 3:**
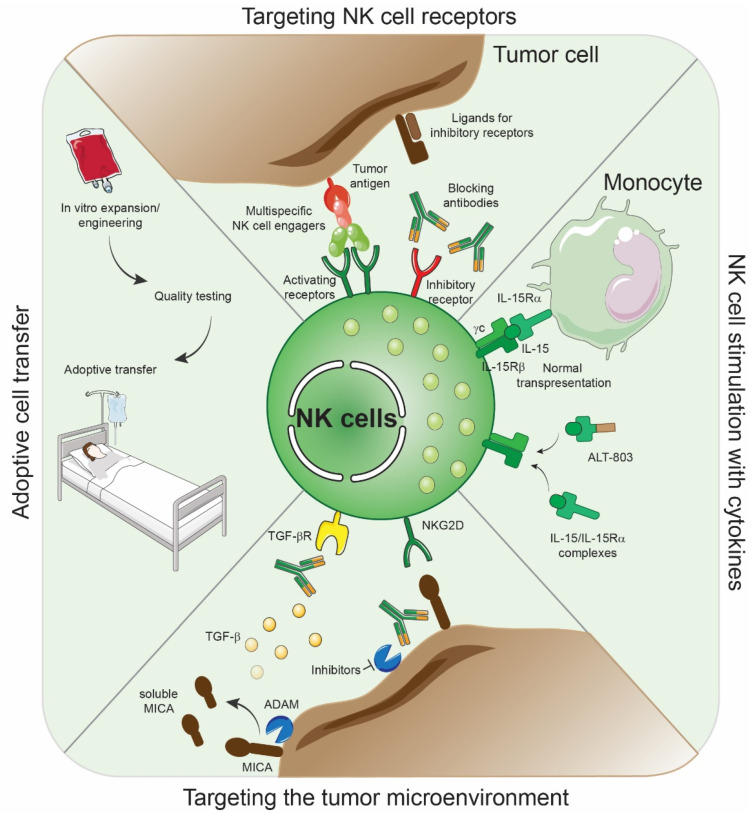
NK cell-directed therapeutic strategies in HCC. Current therapeutic approaches based on NK cell manipulation. Four different strategies are currently being explored. This includes (i) the interference of NK cell receptors to either block the inhibitory receptors or stimulate activating receptors; (ii) the adoptive transfer of autologous/allogeneic NK cells; (iii) the stimulation of NK cells by activating cytokines or (iv) the manipulation of the microenvironment by inhibiting the immunosuppressive pathways found in HCC patients.

NK cells represent a major target to improve cancer immunotherapies. Four major axis are currently widely studied to take advantage of NK cell function in cancer treatment [38,222,223,224,225,226]. These include (i) the production and infusion of engineered NK cells into patients, (ii) the use of agonistic or blocking antibodies targeting NK cells, (iii) NK cell stimulation with cytokines and (iv) the targeting of the tumor microenvironment leveraging NK cell function [222] (Figure 3).

**Adoptive NK cell therapies**. The liver is home to a large proportion of NK cells and, thus, infusion of ex vivo activated/expanded or engineered autologous NK cells may represent a valuable strategy to treat HCC [225]. Despite successes in hematological malignancies, in the context of alloreactive NK cells (mismatch KIR/HLA) [227], the efficacy of adoptively transferred autologous NK cells in solid tumors is, to date, limited. The early encouraging results observed by Rosenberg et al. reinfused autologous lymphokine-activated killer cells together with recombinant IL-2 in metastatic melanoma patients [228]. Additional studies in liver cancer demonstrated superior clinical benefit with the reinfusion of cytokine-induced killer cells plus standard treatment in HCC patients compared to standard treatment alone [229,230]. Furthermore, patients who have received cytokine-induced killer cells as adjuvant therapy, harboring high cytotoxic capabilities, have increased recurrence-free and overall survival compared to control individuals [231,232,233], indicating that cytokine-induced killer cells may represent a suitable therapeutic option to treat HCC patient and to improve disease outcomes [234]. However, in these studies, investigators administered not only NK cells but also other lymphocytes that include T cells, thus, confounding the impact of the two lymphoid cell subsets on patient outcomes. Subsequent clinical trials with purified NK cells were unsuccessful [235], while the infusion of expanded tumor-infiltrating T cells has shown therapeutic benefit in a number of tumor types [236,237,238,239,240,241]. Thus, despite both cell types having a high potential to kill tumor cells, we can question how such differences in clinical outcomes occurred. The absence of ‘conventional’ memory and the lack of antigen-receptor specificity [242] in NK cells could potentially lead to the rapid exhaustion of this pool of ex vivo activated NK cells. In addition, extrinsic factors such as the presence of immunosuppressive factors in the tumor microenvironment may also contribute to inhibit the function of adoptively transferred NK cells. In HCC, high concentrations of TGF-β, indoleamine-2,3-dioxygenase (IDO), prostaglandins and nitric oxide are found in tumors, inhibiting NK cell activity [203,204,205,206,208]. Another explanation could be that infused NK cells do not reach the tumor and thus are unable to mediate their anti-tumor functions. To overcome this, genetically engineered NK cells can be manufactured to express particular chemokine receptors, enhancing their migration capacities towards the tumor bed. Recently, Kremer et al. have demonstrated the improved trafficking of CXCR2 engineered primary NK cells to IL-8 enriched renal cell carcinoma tumor supernatant, and the ability of these NK cells to form increased conjugates with target cells [243]. Other studies have shown the beneficial impact of the infusion of allogenic NK cells associated with irreversible electroporation or cryoablation of liver tumors in advanced HCC patients [244,245]. Lin et al. [244] found that, after 12 days, expanded cultures contained a high proportion of NK cells (CD56^+^CD3^−^), ranging from 71.6% to 96.1%. Patients who have undergone cryoablation treatment associated with the injection of allogeneic NK cells showed increased progression free survival compared to patients who received cryoablation treatment alone [244]. In addition, the investigators observed that multiple infusions of NK cells further improved patient clinical outcomes [244], indicating that increased treatment courses may improve therapeutic responses. Improved HCC patient outcomes were observed in patients receiving irreversible electroporation associated with allogeneic NK cells compared to patients who received irreversible electroporation alone [245], indicating that the transfer of allogenic NK cells could represent a suitable immunotherapy treatment to combine with other HCC therapies, which is deserving of further investigation. Liver cancer cells express several antigens that could be targeted by chimeric antigen receptor (CAR)-modified NK cells [246]. Glypican-3 (GPC3) is an oncofetal protein overexpressed in HCC and associated with poor clinical outcomes [247,248]. Therefore, GPC3 represents a rational target for the development of GPC3-directed CAR-NK cells. Yu et al. [249] transduced the NK-92 cell line with GPC3-specific CAR, composed of an extracellular domain recognizing GPC3 and the CD28 and CD3ζ intracellular and signaling domains. GPC3-specific NK-92/9.28.z cells are functional, produced higher levels of IFN-γ and effectively lysed GPC3^+^ HCC cell lines in vitro, and significantly reduced the tumor growth of GPC3^+^ HHC in vivo [249]. Similar findings were reported with CAR NK cells against c-MET or CD147, two rational targets in HCC [250,251,252,253]. TGF-β accumulates in HCC, and its signaling inhibits NK cell function and anti-tumor immunity. With this in mind, Wang et al. [254] ingeniously engineered the NK-92 cell line to express a chimeric receptor composed of the TGF-βRII extracellular and transmembrane domains associated with the intracellular domain of NKG2D. Thus, in this setting, the engagement of TGF-βRII induced NK cell activation and function. These CAR NK cells were resistant to TGF-β-induced suppression and NKG2D downregulation [254]. In addition, they exhibited improved IFN-γ production and cytotoxicity against HCC cell lines in vitro and enhanced control of HCC tumor growth in vivo [254]. Importantly, the authors observed increased NK cell homing to TGF-β^+^ tumors [254], offering a sound strategy to increase NK cell migration to tumors. Collectively, these findings suggest that the adoptive transfer of NK cells or engineered CAR-NK cells could represent a suitable therapeutic option for HCC patients and warrant further investigation. To this end, a number of clinical trials are currently evaluating the safety and efficacy of NK cell infusion as monotherapy or in combination with standard treatment or ICB in HCC patients (Table 3).

**Targeting NK cell receptors.** Many strategies to engage activating receptors or to block the inhibitory receptors-ligands are currently being evaluated in order to increase NK cell function in cancer [255,256]. The use of a blocking antibody against NKG2A [257] or an NKG2A protein expression blocker [258], engineered to abrogate NKG2A expression by preventing its translocation to the cell membrane, have proven to be therapeutic in preclinical mouse models, overcoming NK cell resistances, but remain to be tested in HCC. Other approaches consisting of targeting the KIR family using monoclonal antibodies or a peptide:MHC DNA vaccine have shown promising results in preclinical mouse models and in non-HCC tumor types [259,260,261,262,263], deserving further development and testing in liver cancer. Based on preclinical studies and observations made in HCC tumors, the targeting of other NK cell inhibitory receptors such as TIGIT [264], Siglec-7 [265] or LAG3 [266,267], represents suitable therapeutic options that warrant further investigation. Conversely, the development of multispecific NK cell engagers, composed of fragments of monoclonal antibodies and designed to stimulate NK cell function, has gained insights and represent valuable candidates to enhance NK cell anti-tumor activity [268]. Particularly, these molecules increase NK cell-tumor cell interactions by targeting an NK cell activating receptor and a tumor antigen. These result in increased NK cell cytotoxicity and improved anti-tumor responses in preclinical models [269,270,271], as well as increased NK cell function and response rates in early clinical trials [272,273]. Together, NK cell-based immunotherapeutic options are currently being developed and tested in preclinical mouse models and in clinical studies, with the potential to become the next wave of immunotherapeutics in cancer.

**NK cell stimulation with cytokines**. Intratumoral hepatic NK cells are dysfunctional. However, in vitro cultured tumor-infiltrating NK cells with IL-15 overcome resistances and restore NK cell function and anti-tumor activity in HCC [274]. These results suggest that harnessing IL-15 signaling in NK cells represents another suitable option to further enhance disease outcomes [275]. Although IL-15 is used to maintain activated NK cells during ex vivo expansion, the direct infusion of IL-15 in patients remains limited due to its short half-life [276]. Considerable efforts are currently being made to develop and test the activity of modified IL-15, such as IL-15/IL-15Rα complexes, in extending IL-15 biological function [277] without the need for IL-15 trans-presentation by IL-15Rα expressing cells [278,279]. These complexes enhance NK cell development, differentiation and function [280,281,282,283]. The efficacy of such complexes is currently being evaluated in clinical trials (Table 3). Nevertheless, these complexes also induce toxicities that are mainly attributed to IFN-γ production by NK cells [280]. Other approaches consist of coupling IL-15 to the sushi domain of the IL-15Rα that is fused to and IgG-Fc fragment antibodies to increase its stability. The IL-15 supplementation to NK cells using this molecule, ALT-803, demonstrated improved anti-tumor outcomes in preclinical models [284,285,286,287,288] and phase I clinical trials [289]. Given these promising results, such treatments are currently being tested in HCC, aiming to reverse NK cell dysfunction and enhance patient outcomes (Table 3).

**Targeting the tumor microenvironment.** TGF-β induces CD96 and reduces CD226 expression on NK cells [139], a phenotype associated with reduced IFN-γ production. The plasma of HCC patients is enriched in TGF-β. NK cells cultured in vitro in the presence of plasma from HCC patients resulted in NK cell dysfunction. However, blocking TGF-β signaling using antibodies restores NK-cell IFN-γ production [139], indicating that targeting TGF-β pathways represents a suitable option to reinvigorate NK cell function and treat HCC patients [290,291,292]. The treatment with sorafenib triggered NK cell activation in tumor bearing mice [293]. Mechanistically, this TKI rewired TAM and promoted a pro-inflammatory phenotype consisting of IL-12 production, which then activated NK cell degranulation and IFN-γ production upon target cell contact [293]. Consequently, the combination of sorafenib with NK cell-directed immunotherapies may further improve clinical outcomes in HCC [294]. The therapeutic targeting of the proteases responsible for the shedding of NKG2D ligands from the surface of tumor cells represents an additional valuable strategy to reinstate NK cell anti-tumor function and improve patient prognosis. In vitro studies have identified several inhibitors with significant potential to inhibit ADAM9 and/or ADAM10 function that can restore NK cell function [210,295]. In addition, the treatment of a HCC cell line with sorafenib diminished ADAM9 expression, leading to reduced MICA shedding [212]. As a consequence, increased MICA expression at the surface of HCC cell lines are detected, increasing NK cell-mediated tumor cell lysis in an NKG2D-MICA dependent manner [212]. Furthermore, decreased ADAM17 expression in HCC cell lines was observed after treatment with the specific inhibitor TMI-1, resulting in increased membrane bound MICA and a decrease of its soluble form [213]. Arai et al. [213] performed an in vitro screen to determine additional inhibitors of ADAM17. They found that the treatment of HCC cell lines in vitro with lomofungin, an FDA-approved antibiotic compound, significantly decreased ADAM17 expression, increasing membrane MICA and thus, decreasing sMICA in culture media [213]. Given the critical roles played by the ADAM proteases in homeostatic development and cell growth, such inhibitors must be carefully used, and the generation of analogs with specific targeting properties has to be developed and tested using preclinical models. Alternatively, the use of antibodies targeting the site of proteolytic shedding prevents the loss of MICA/B surface expression by human tumor cell lines, inhibiting tumor growth and metastases in humanized mouse models [296]. Such strategies have yet to be tested in HCC, but strong anti-tumor responses may be expected.

## 9. Conclusions and Future Perspectives

The targeting of the immune system represents a therapeutic revolution that has fundamentally changed the clinical management and prognosis of cancer patients. However, the success of these treatments, thought to be mainly based on the reactivation of T cell anti-tumor functions, may not be relevant for every tumor type. In HCC, the overall response rates remain modest, and only a minority of patients exhibit durable responses to the combination atezolimumb–bevacizumab. Thus, alternative treatments are needed to improve the clinical prognosis of this disease. It is clear that innate cells, particularly ILCs, offer a number of advantages in cancer treatment. They provide early critical mediators to mount potent anti-tumor responses (i.e., XCL1) and exert critical cytotoxic function to eliminate cancer cells. However, these cells are often dysregulated in the tumor microenvironment, displaying an immunoregulatory and exhausted phenotype. Novel approaches are currently being investigated to overcome ILC dysfunction and to exploit their potential. Although the targeting of ILCs holds great promise, a better understanding of their function, prognostic value and therapeutic potentials are still needed.

Many important questions remain to be answered. In humans, what is the role and function of tissue resident hepatic ILC1, as identified by Forkel et al. [57]? Given the heterogeneity of hepatic NK cells, increased resolution of scRNA sequencing analyses is required to further ascertain the different characteristics of hepatic NK cell subsets. Then, subsequent validation at the protein level and functional ex vivo assays will be necessary. The liver is an immunotolerant organ, and both intrinsic and extrinsic mechanisms are responsible for NK cell dysfunction. Therefore, it is critical to not only investigate the NK cell phenotype and function, but also to comprehensively analyze parenchymal and non-parenchymal populations to identify suitable pathways for future targeting.

Important differences were observed in liver group 1 ILC composition and function between mice and humans. Thus, we can ask whether preclinical mouse models are suitable to study the role and function of NK cells and ILC1 in HCC development and treatment efficacies. In addition, despite substantial progress in conditional gene deletion, targeting ILC1 has not yet been achieved with the current models [297]. Given the large numbers of receptors and ligands, and transcription factors shared between NK cells and ILC1, this poses difficulties in finding a specific target for ILC1. Using conditional gene targeting in cells expressing *Ncr1* (encoding NK-46) [298,299,300], specific deletion of *Eomes* in NKp46 expressing cells abolished NK cell development and maturation, but not ILC1 [90,91]. Thus, this mouse model is suitable to study the impact of NK cells but not of other NKp46-expressing ILCs such as ILC1. Another strategy has been to cross *Tbx21^fl/fl^* mice with *NKp46^Cre/+^* mice, aiming to delete ILC1 [69,70,87]. ILC1 development requires T-bet expression, but is also required for cNK cell maturation and function [70,301]. More recently, Bai et al. have deleted hepatic ILC1 by targeting the IFN-γ signaling pathway. *NKp46^Cre/+^Ifng^fl/fl^* mice harbor a specific deletion of liver ILC1. However, when investigating the function of the cells in HCC development, the impairment of IFN-γ production by other NKp46 expressing cells, and notably cNK cells, will certainly influence disease outcomes, confounding the effect of ILC1 on cancer development and progression. Thus, so far, there are no specific mouse models to precisely investigate the role of liver ILC1 in HCC development and progression. Recent investigations using preclinical models of liver metastasis suggest that ILC1 are essential to control metastatic seeding, whereas NK cells restrain tumor outgrowth [302].

Finally, the safety and clinical efficacy of newly developed NK cell-directed therapies such as multispecific NK cell engagers, remains to be fully evaluated [269,303,304]. Further investigations are warranted to determine the best suitable therapeutic regime and whether combination therapies involving NK cell-targeted treatments further improve anti-tumor responses and clinical outcomes. Based on recent findings, in addition to multispecifc NK cell engagers, blocking the inhibitory receptors NKG2A, TIGIT, LAG3 or KIRs expressed on NK cells using antibodies represents a potential therapeutic option, which deserves further investigation in HCC.

## Figures and Tables

**Table 1:** Phenotypic and functional characteristics of human and mouse hepatic NK cells and ILC1.

	Phenotypic Markers			Effector Molecules	References
	Mouse	Human			
		cNK Cells	lrNK Cells		
NK cells	NK1.1^+^, NKp46^+^, CD49a^−^, Eomes^+^, T-bet^+^, CD49b^+^, CD200R^−^, Ly49^+/−^, CXCR6^−^, Killer cell lectin-like receptor G1(KLRG1)^+/−^, CD62L^+/−^	CD56^dim/bright^, CD16^bright/low^, NKG2D^+/−^, NKp46^+^, CD49b^+^, CD49e^+^, CD200R^−^, Eomes^+^, T-bet^+^	CD56^+^, CD16^−^, NKG2D^+^, NKG2C^+^, NKG2A^−^, NKp46^+^, CD49e^−^, CXCR6^+^, CD69^+^, Eomes^hi^, T-bet^−^	Granzyme A and B, Perforin, IFN-γ	[55,60,61,62,66,67,68,69,70,71,72]
ILC1	NK1.1^+^, NKp46^+^, CD49a^+^, T-bet^+^, TRAIL^+^, CD49b^−^, CD200R^+^, CD62L^−^, KLRG1^−^, CXCR6^+^, CD127^+^	Lineage^−^CD127^+^CD16^−^NKG2A^−^NKp44^−^CD117^−^		Granzyme B^+^, interleukin (IL)-2, IFN-γ, Tumor necrosis factor (TNF)-α, granulocyte-macrophage colony-stimulating factor (GM-CSF)	[55,57,69,70,71,72,73]

**Table 2:** Consequences of the loss of key transcription factors on liver ILC1 and NK cell homeostasis in mice.

Targeted Gene	Liver NK Cells	Liver ILC1	References
*Tbx21*	Strongly reduced	Absent	[69,70,87]
*Eomes*	Absent	No impact	[90,91]
*Nfil3*	Absent	Reduced or no impact	[69,71,93,94]
*Hobit*	No impact	Absent	[73]

**Table 3:** Strategies currently evaluated in clinical trials, consisting of taking advantage of NK cell-based immunotherapies in HCC.

Treatment Strategy	Allocation	Outcomes Measured	Clinical Trials.gov Identifier
FT500, an allogeneic NK cell product, as monotherapy and in combination with IL-2 and/or immune checkpoint inhibitors (anti-PD_1 and anti-PD-L1)	Non-Randomized	Primary outcome measures: Dose Limiting toxicities Secondary outcome measures: Objective-response rates (ORR) and pharmacokinetics of FT500 in blood	NCT03841110
A Phase II/III clinical trial withe ex vivo expanded autologous immune killer cells to treat liver cancer patients as an adjunct therapy	Randomized Autologous NK cells + transcatheter arterial chemoembolization (TACE) vs. TACE alone	Primary outcome measures: Change of tumor size and progression-free survival (PFS) Secondary outcome measures: Promotion of anti-cancer immune responses	NCT03592706
Safety and efficacy of allogeneic NK cell therapy in patients with advanced hepatocellular carcinoma	Non-Randomized	Primary outcome measures: Incidence of Adverse events and Overall survival (OS) Secondary outcome measures: Disease control rate	NCT04162158
FATE-NK100 as monotherapy and in combination with monoclonal antibody in subjects with advanced solid tumor	Non-Randomized	Primary outcome measures: Dose Limiting toxicities Secondary outcome measures: ORR and pharmacokinetics of FATE-NK100 in blood	NCT03319459
By using adoptive transfer of autologous NK cells to prevent recurrence of hepatocellular carcinoma after curative therapy	Randomized NK cell infusion + curative therapy (stage I and II patients only) vs. curative therapy only	Primary outcome measures: Recurrence free survival and OS	NCT02725996
Study of SO-C101 (IL-15 superagonist) and SO-C101 in combination with pembrolizumab in adult patients with advanced/metastatic solid tumors	Non-Randomized	Primary outcome measures: Dose Limiting toxicities and adverse events Secondary outcome measures: Pharmacokinetics, ORR, best overall response, duration of response (DOR), clinical benefit rate, PFS and immunogenicity	NCT04234113
QUILT-3.055: A study of combination immunotherapies (N-803, an IL-15 superagonist + ICB) in patients who have previously received treatment with immune checkpoint inhibitors	Non-Randomized	Primary outcome measures: ORR Secondary outcome measures: Disease-specific survival, OS, time to response, DOR, incidence of adverse events, quality of life and PFS	NCT03228667
